# Anti-ADAMTS13 Autoantibodies in Immune-Mediated Thrombotic Thrombocytopenic Purpura

**DOI:** 10.3390/antib14010024

**Published:** 2025-03-10

**Authors:** Michael R. Snyder, Robert W. Maitta

**Affiliations:** Department of Pathology, University Hospitals Cleveland Medical Center, Case Western Reserve University School of Medicine, Cleveland, OH 44106, USA; michael.snyder@uhhospitals.org

**Keywords:** ADAMTS13, autoantibody, von Willebrand factor, thrombotic thrombocytopenic purpura, iTTP, structure, specificity, susceptibility

## Abstract

Autoantibodies to ADAMTS13 are at the center of pathology of the immune-mediated thrombotic thrombocytopenic purpura. These autoantibodies can be either inhibitory (enzymatic function) or non-inhibitory, resulting in protein depletion. Under normal physiologic conditions, antibodies are generated in response to foreign antigens, which can include infectious agents; however, these antibodies may at times cross-react with self-epitopes. This is one of the possible mechanisms mediating formation of anti-ADAMTS13 autoantibodies. The process known as “antigenic mimicry” may be responsible for the development of these autoantibodies that recognize and bind cryptic epitopes in ADAMTS13, disrupting its enzymatic function over ultra large von Willebrand factor multimers, forming the seeds for platelet activation and microthrombi formation. In particular, specific amino acid sequences in ADAMTS13 may lead to conformational structures recognized by autoantibodies. Generation of these antibodies may occur more frequently among patients with a genetic predisposition. Conformational changes in ADAMTS13 between open and closed states can also constitute the critical change driving either interactions with autoantibodies or their generation. Nowadays, there is a growing understanding of the role that autoantibodies play in ADAMTS13 pathology. This knowledge, especially of functional qualitative differences among antibodies and the ADAMTS13 sequence specificity of such antibodies, may make possible the development of targeted therapeutic agents to treat the disease. This review aims to present what is known of autoantibodies against ADAMTS13 and how their structure and function result in disease.

## 1. Introduction

Thrombotic thrombocytopenic purpura (TTP) is a rare and potentially fatal thrombotic microangiopathy (TMA). The pathophysiology of TTP involves the formation of platelet-rich microthrombi, which occlude the microvasculature and cause end-organ ischemia and injury [1,2,3]. The acquired form of the disease, known as immune-mediated thrombotic thrombocytopenic purpura (iTTP), is caused by autoantibodies against the ADAMTS13 enzyme (a disintegrin and metalloproteinase with a thrombospondin type 1 motif, member 13), whose function is proteolysis of von Willebrand factor (vWF) multimers. Decreased ADAMTS13 activity caused by autoantibody binding leads to a buildup along endothelial walls of ultra large vWF multimers, which bind platelets inappropriately under conditions of high shear stress. The pathologic role that these anti-ADAMTS13 autoantibodies play in the pathogenesis of iTTP has become a growing area of research. Therefore, understanding the structure and function relationships of these autoantibodies, as well as their mechanisms of interaction with ADAMTS13, and specifically what triggers their formation can improve our knowledge of the pathophysiology of iTTP. This will prove helpful in guiding therapy, developing new treatments, establishing disease susceptibility, and hopefully chart new areas of investigation.

As with other TMAs, such as Hemolytic Uremic Syndrome (HUS), iTTP leads to the characteristic clinical findings of severe thrombocytopenia, hemolytic anemia due to intravascular hemolysis, and end-organ damage [4]. Ischemic end-organ injury typically manifests as neurological symptoms such as cerebrovascular accidents, seizures, headaches, altered mental status, and psychosis, as well as acute kidney injury. The role of ADAMTS13 deficiency in the pathogenesis of iTTP was first described in the late 1990s [5] and was soon followed by identification of the causative role of anti-ADAMTS13 antibodies in precipitating disease onset [6]. Currently, while clinical symptoms, hemolysis labs, and the presence of fragmented erythrocytes on peripheral blood smear can increase clinical suspicion, disease confirmation requires ADAMTS13 activity levels at <5–10% of normal at presentation. A very low level of ADAMTS13 activity can help to distinguish between iTTP and other TMAs or TMA-related conditions, such as HUS, sepsis, and liver failure, as well as pregnancy-related TMAs, such as disseminated intravascular coagulation and preeclampsia with HELLP syndrome [7,8]. Pregnancy therefore represents a special diagnostic challenge since it is also a known trigger of iTTP [9]. Compounding this, pregnancy is also the time when the congenital form of the disease (cTTP) can first manifest itself [10]. ADAMTS13 activity can be decreased in some of these conditions, as low as 10–20% of normal, but usually not to the extent found in iTTP. Accurate diagnosis is important because therapeutic plasma exchange (TPE) is the gold standard for iTTP treatment, as circulating autoantibodies are removed from the plasma while ADAMTS13 stores are replenished. Notably, ADAMTS13 inhibitor levels may also be measurable and help distinguish between iTTP and cTTP since the latter would lack the presence of autoantibodies.

In light of the autoimmune nature of iTTP, the aim of this review is to present the immune mechanisms and immune dysregulation leading to disease presentation. This includes looking at formation of autoantibodies against ADAMTS13 in the context of potential immune triggers for this antibody formation, including processes such as antigenic mimicry, immune susceptibility in the population, and the presence of auto-reactive immune cells. Autoantibody structure and function relationships will be introduced, including the importance of conformational changes to ADAMTS13 in mediating antibody binding, the formation of antigen–antibody immune complexes, and interactions among the antibodies of different mechanisms, including inhibitory and non-inhibitory. This will be contrasted with current testing methodologies and what future research is currently heading towards. All of this will underscore the complexity of anti-ADAMTS13 autoantibodies while providing a clearer picture of disease and, importantly, presenting opportunities for the future development of targeted therapies.

## 2. Formation and Structure of Anti-ADAMTS13 Antibodies

### 2.1. Molecular Mimicry and Antibody Pathogenesis

Formation of iTTP autoantibodies is due to immune system dysregulation mediated by a complex interplay between genetic and environmental factors. Viral and bacterial infections, including HIV, Hepatitis, Influenza (A), and, recently, COVID-19 have all been implicated in triggering iTTP via a process of molecular antigenic mimicry [11,12,13]. Molecular mimicry has been well studied in other diseases such as infections with polysaccharide-encapsulated microorganisms, in which peptides can mimic polysaccharide antigenic determinants and elicit anti-polysaccharide antibodies [14]. This type of mimicry occurs when T lymphocytes are primed by the antigenic determinants of these pathogens and then cross-react with peptides derived from ADAMTS13, which may be conformationally similar in amino acid composition or in structure to pathogen-derived peptide sequences [15,16]. Specifically, peptide elution studies of the ADAMTS13 peptidome presented by human leukocyte antigen (HLA) Class II molecules have detected numerous 9-mer core peptide sequences. These are derived primarily from the C1r/C1s-Uegf-Bmp1 (CUB) 1 and CUB 2 domains of ADAMTS13 and include FINVAPHAR (HLA-DR) and ASYILIRD (HLA-DQ) [15], among others. Studies of patients with iTTP have found evidence of FINVAPHAR-reactive and ASYILIRD-reactive CD4^+^ T cells [17], highlighting the possible role of these specific peptide sequences in mimicking microbial-derived peptides. These peptides have in fact been described as immunologically “promiscuous”, since, in elution studies they bind non-selectively to different HLA Class II molecules, a trait that may contribute to their cross-creativity in vivo. This process of molecular mimicry is further mediated by pattern recognition receptors (PRRs), signaling proteins on dendritic cells and other antigen-presenting cells (APCs) that recognize pathogen-associated molecular patterns, leading to subsequent immune system upregulation [18]. Notable PRRs include Toll-like receptors (TLRs), which, when activated by specific microbes, increase levels of co-stimulatory molecules, such as CD40 and CD80, on the surface of APCs.

Molecular mimicry has been associated with a wide variety of autoimmune diseases, including systemic lupus erythematosus (SLE), systemic sclerosis, and rheumatic fever [19,20,21]. In the case of SLE, molecular mimicry is thought to occur after infection with Epstein–Barr virus (EBV) results in the formation of EBV nuclear antigen-1, against which autoantibodies cross-react with self-antigens linked to SLE, such as the Ro/SSA ribonucleoprotein [22]. However, in the case of iTTP, the virus-specific peptides and mimotopes undergirding host immune system reactivity have yet to be discovered. This task is undoubtedly complicated by the wide range of documented infections and inflammatory triggers for iTTP initiation. Additionally, case reports have also implicated certain pharmaceutical drugs in the development of iTTP, including clopidogrel and ticlopidine [23,24]. In these cases, autoimmunity may result from the cross-reactivity of antigenic drug byproducts following metabolism by the liver, such as protein adducts that resemble self-antigens [25].

### 2.2. Role of T and B Lymphocytes in Autoimmune Response

Dysregulated T and B lymphocyte activation plays a critical, yet still poorly understood, role in the etiology of a systemic autoimmune response in iTTP. As described above, ADAMTS-13-derived peptides are loaded onto the MHC-class II molecule expressed on the surface of dendritic cells and other APCs, which then presents them to T cells. Loss of tolerance is thought to emerge from a small number of T lymphocytes escaping the normal process of negative selection and apoptosis in the medulla of the thymus, possibly due to altered T-cell receptor affinity to self-antigens [26]. For reasons that are not well understood, these T cells nevertheless become sufficiently activated at extrathymic locations when they encounter MHC-class II/self-peptide complexes, leading to autoimmune responses. Even though this could represent an alternative source of ADAMTS13 auto-reactive T cells, this cell population would be inherently distinct from those cells, which respond to foreign antigenic determinants but cross-react with a self-antigen. Once activated by APCs, CD4^+^ T-Helper cells activate B lymphocytes to proliferate in secondary lymph nodes. These B lymphocytes ultimately differentiate into either antibody-producing plasma cells or memory B cells, which respond with antibody production when re-exposed to the eliciting antigen at a later time. The B-cell phenotype is known to be altered in iTTP, specifically in terms of reported changes in the frequency of CD80 expression, a co-stimulatory receptor that is required for sustained T-cell activation and humoral immune response [27]. Expression of CD80, which is upregulated in other autoimmune diseases, such as SLE, is likewise found to be increased in the plasmablasts (plasma cell precursors) of patients with iTTP [28]. Compared to heathy controls, the overall numbers of plasmablasts were increased, post-germinal center memory B cells were decreased, and T-Helper cells were decreased in the circulation, all of which are suggestive of dysregulated homeostasis between T and B lymphocytes in iTTP.

### 2.3. Haplotype and Genetic Susceptibility to Autoimmunity

It has been proposed that autoantibody formation occurs preferentially in individuals with specific HLA alleles. This suggests that there is a genetic predisposition to the disease. For example, Caucasian patients expressing the HLA-DRB11*1 and HLA-DQB1*02:02 haplotypes have been reported to be at significantly higher risk for iTTP [29,30]. These HLA-class II regions on APCs, when presenting ADAMTS-13-derived peptides, appear to be preferentially recognized by self-reactive T cells [31]. By contrast, individuals with HLA-DRB1*04 and HLA-DRB4 haplotypes are decreased in iTTP patients, suggesting that there is a possible protective effect from disease by these haplotypes [32]. Importantly, this susceptibility may not be restricted to a particular ethnicity, since iTTP is seen across populations [33]. In addition to shared haplotype, the co-occurrence of a wide variety of autoimmune manifestations in patients with iTTP may point to common genetic determinants in this affected population [34]. Severe ADAMTS13 deficiency has been predominantly detected in patients with SLE and rheumatoid arthritis (RA); however, systemic sclerosis, polymyositis, sarcoidosis, autoimmune thyroiditis, and Reynaud phenomenon have all been variously described in association with iTTP. One study found that 71% of patients with severe ADAMTS13 deficiency were also positive for anti-nuclear antibodies, with several others possessing anti-double stranded DNA antibodies and anticardiolipin antibodies [35]. While familial clustering has not been extensively described (apart from the autosomal recessive form of the disease, Upshaw–Schulman syndrome, caused by ADAMTS13 gene mutations), several case reports have documented first-degree relatives affected with the immune form of the disease [36,37]. This is an area in which future and larger studies may establish such familial links of the disease.

### 2.4. Structure of Anti-ADAMTS13 Antibodies

IgG antibodies are the main antibody class detected in the plasma of patients with iTTP. Of these, IgG4 is the most prevalent subclass; however, IgG1, IgG2, and IgG3 have also been detected [38]. IgG1 is the dominant subclass during the first acute episode, while IgG4 appears more commonly during relapsed iTTP [38,39]. Interestingly, there appears to be an inverse correlation between the frequency of IgG1 and IgG4 antibodies found in the plasma in iTTP patients [39]. Less commonly, IgM and IgA isotypes have also been identified [40]. Notably, during immunoglobulin rearrangement, the VJD recombination of heavy chains generates an antibody’s variable region, which comprises unique antigen receptors; however, anti-ADAMTS13 antibodies preferentially incorporate heavy chain gene segment VH1-69, a prevalence likely explained by its complementary binding with exosites on the ADAMTS13 enzyme [41].

## 3. Mechanisms of Antibody Interaction with ADAMTS13

**Figure 1:**
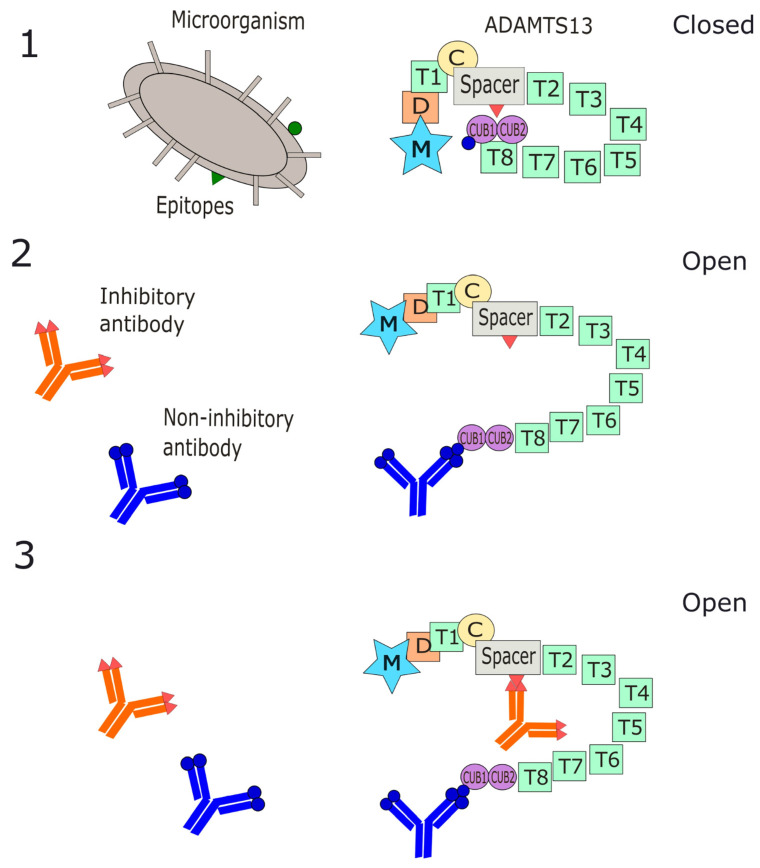
A working model of anti-ADAMTS13 autoantibody formations and mechanisms of binding in iTTP. (**1**) Under physiologic conditions, ADAMTS13 circulates in a folded or “closed” conformation mediated by interactions between its central spacer domain and CUB domains. Initial autoantibody generation is thought to occur due to “molecular mimicry”. This process occurs when T lymphocytes are primed by antigenic determinants of pathogens (green triangle, green circle), which cross-react with peptides/epitopes from ADAMTS13 (red triangle, blue circle), conformationally similar in amino acid composition or in structure to pathogen-derived peptide sequences. (**2**) Under pathologic conditions, possibly triggered by re-exposure to antigenic determinants later during infection, inflammation, or a yet-to-be-defined stressor, non-inhibitory autoantibodies may be generated and bind to the distal C-terminal domain or other domains. This forces a conformational change in ADAMTS13 into its less stable “open” conformation, which alone may be sufficient to cause ADAMTS13 dysfunction and disease in some patients. (**3**) In some patients, exposure to cryptic epitopes (red triangle) in the central spacer domain during this “open” state facilitates the binding of specifically neutralizing antibodies, which directly inhibit ADAMTS13 enzymatic function. Decreased ADAMTS13 activity leads to a buildup of vWF multimers along endothelial walls, resulting in the formation of platelet-rich microthrombi. M = metalloprotease domain; D = disintegrin-like domain; T1-8 = thrombospondin type 1 repeats; C = cysteine-rich region; Spacer = spacer domain; CUB 1 and 2 = C1r/C1s-Uegf-Bmp1 domains.

The ADAMTS13 enzyme is a zinc metalloprotease consisting of 1427 amino acids with a molecular weight of 190 kD [42,43]. The enzyme consists of 14 domains, including a metalloprotease domain, a disintegrin-like domain, eight thrombospondin type 1 repeats, a cysteine-rich region, a spacer domain, and two complement CUB domains (Figure 1) [42]. Epitope mapping of ADAMTS13 has revealed a polyclonal antibody response during acute iTTP. The majority of autoantibodies are directed against the central cysteine-rich spacer region [44,45,46,47,48]. While most iTTP patients demonstrated anti-cysteine-spacer antibodies, antibodies directed against other domains of ADAMTS13 have been described [46]. These include CUB domains, thrombospondin type 1 domains, and C-terminal domains. Importantly, multiple autoantibodies with different specificities have been detected simultaneously in the same patient [49].

The cysteine-rich spacer domain has been identified as an essential exosite for the proteolytic activity of vWF [50]. The anti-cysteine-spacer antibodies react with three so-called “hotspots” in the cysteine-spacer domain, corresponding primarily to amino acid regions 588–592, 602–610, and 657–666 [51]. Specifically, a single epitope comprising Argenine568, Phenylalanine592, Arginine660, Tyrosine661, and Tyrosine665 on the outer surface of the cysteine-spacer domain is a frequent target of anti-ADAMTS13 IgG antibodies [52]. These residues are known to play an essential role in the binding of ADAMTS13 to vWF in healthy patients [53].

In its inactive state, ADAMTS13 circulates in a folded or “closed” conformation, mediated by interactions between the spacer and CUB domains. A hallmark of the acute iTTP presentation is adoption of an “open” conformation following disengagement of the spacer and CUB domains [54]. This is an apparent contradiction, since the “open” conformation is also the allosterically active conformation during which proteolytic activity of vWF is possible. However, under physiologic conditions, ADAMTS13 opens only transiently, allowing short-lived enzymatic interactions with ultra large vWF multimers before returning to its more stable “closed” state [55]. Therefore, the persistence of this “open” conformation in iTTP, in the absence of endothelial injury, is considered less stable and is ultimately associated with enzymatic dysfunction. Of note, ADAMTS13 returns to a folded position once iTTP remission is achieved (Figure 1) [56]. The mechanisms by which ADAMTS13 becomes “locked” into this open conformation during iTTP are not yet understood, but various triggers have been hypothesized. These include prolonged conditions of high shear stress or a massive release of vWF in response to infection, inflammation, or another yet-to-be-defined stressor [8,57]. Yet another important mechanism for our purposes is the binding of non-neutralizing autoantibodies, as described in the next section.

## 4. Function and Effects of Anti-ADAMTS13 Antibodies

As mentioned above, anti-ADAMTS13 antibodies are polyclonal, but they exert their effects via both inhibitory and non-inhibitory mechanisms [58,59]. Inhibitory antibodies lead to inhibition of the proteolytic activity of ADAMTS13. Anti-spacer antibodies have been identified as the major inhibitory antibodies responsible for neutralizing ADAMTS13 activity, although it should be noted that not all anti-spacer antibodies are inhibitory. Recent immunoprofiling has revealed that the majority of patients (~73%) will have anti-spacer antibodies [49,60]. By contrast, non-inhibitory antibodies result in depleted levels of ADAMTS13 antigen without directly neutralizing its enzymatic activity. In fact, many patients with iTTP lack significant inhibitory antibodies and instead have depleted levels of ADAMTS13 protein [48]. Antibodies solely targeting the C-terminal of the protein have been among those shown to be of a non-inhibitory nature [61]. Among non-inhibitory antibodies, so-called “clearing” antibodies are believed to accelerate ADAMTS13 clearance from circulation [62,63]. This is thought to occur in part via the formation of ADAMTS13 antigen–antibody immune complexes. These complexes have been detected in a subgroup of patients with iTTP, and these can potentially persist in circulation for years [64,65].

The binding of anti-ADAMTS13 antibodies is highly conformation-dependent and increases when ADAMTS13 is in the “open” conformation [66]. This is thought to occur because the “open” conformation reveals antigenic binding sites, or cryptic epitopes, in the cysteine-spacer region. These cryptic epitopes are subsequently recognized by certain iTTP autoantibodies [67]. However, some autoantibodies can target “closed” ADAMTS13 as well [66]. Along these lines, antibodies have been detected which induce ADAMTS13 to transition from a “closed” to an “open” conformation upon binding [68]. For example, anti-cysteine/spacer and anti-CUB antibodies have been found to have an ”opening” effect on ADAMTS13 during acute iTTP presentations [69]. Likewise, antibodies binding the distal C-terminal have also been shown in vitro to stimulate ADAMTS13 activity, possibly by inducing a persistent “open” conformation [70].

## 5. Anti-ADAMTS 13 Antibodies and Clinical Correlates

Measurements of anti-ADAMTS13 antibodies are emerging in clinical trials as a key prognostic factor in determining iTTP severity, mortality, and possible recurrence. Increasing levels of antibodies are generally correlated with increased burden of disease. Specifically, higher titers of IgA, IgG1, and IgG3 have been associated with more severe iTTP episodes [71]. Increased IgG antibodies have also correlated strongly with mortality, as well as increased cardiac and neurological involvement [72]. Median inhibitory antibody titers at the time of diagnosis have likewise been associated with the total number of TPE procedures required to achieve clinical response and remission [73]. Furthermore, patients with autoantibodies targeting ≥6 domains of ADAMTS13 appear to be at higher risk of death, although this occurred in only 5.6% of patients in one cohort [60]. Patients with anti-metalloprotease antibodies had significantly worse thrombocytopenia than patients without anti-M antibodies. Of note, approximately 36% of patients will possess only a single antibody, usually anti-spacer [73]. By contrast, patients with no detectable inhibitory antibodies have had a more rapid and lasting response to treatment, suggesting that there is an inverse correlation between antibody levels and treatment responsiveness that also depends upon the quality of the antibody [74].

Theoretically, the greater risk in patients with antibodies with multiple specificities compared to those with mono-specificity could be due to the position of the epitopes that drove antibody production. In this model, the enzyme’s conformational change drove the production of antibodies in the first place, so folding of the enzyme into its more stable, inactive form would not necessarily be sufficient for preventing the binding of poly-specific antibodies or concealing all the antigenic determinants that resulted in the generation of antibodies, thus making disease more difficult to treat. Such patients would also be more likely to relapse since those antigens could drive antibody responses more readily at a later time depending upon their protein location. Studies are needed to establish if, indeed, patients with antibodies with multiple specificities are more relapse-prone.

In terms of recurrence risk, the likelihood of iTTP recurrence increased 3.6 times during remission in patients possessing both severe ADAMTS13 deficiency and anti-ADAMTS13 antibodies, regardless of their neutralizing activity [75]. Importantly, there also appears to be a statistically significant association with disease relapse for patients with anti-ADAMTS13 antibodies >15 U/mL, which was confirmed at 3 and 6 months of follow-up [76]. According to this study, patients with both increased anti-ADAMTS13 antibody titers and ADAMTS13 activity <20% had a 2-fold increase in relapses over this period. Likewise, elevated IgG titers have been specifically associated with increased recurrence risk [71]; however, another study revealed that, while decreased ADAMTS13 activity was associated with a higher risk of relapse, IgG antibody levels were not by themselves predictive of relapse within 3 months of specimen collection [77]. Lastly, the presence of ADAMTS13-circulating immune complexes has also been associated with the risk of recurrence within 2 years after the first iTTP episode, but not with overall disease severity [78].

## 6. Laboratory Detection of Anti-ADAMTS 13 Antibodies

ADAMTS13 activity testing is at the center of the diagnosis of iTTP. As mentioned earlier, an activity of <5–10% is recognized as being indicative of such a diagnosis [79]. Of the tests currently available, the fluorescence resonance energy transfer (FRETS)-VWF73 assay is still considered the gold-standard since being first described twenty years ago [80]. This test measures the cleavage of a synthetic vWF peptide which, in the presence of physiologic concentrations of ADAMTS13, leads to quantitative increases in fluorescence but does not change signaling in the absence of the enzyme. Despite its relative ease of use, it is heavily operator-dependent, and, as a result, these tests tend to be performed at reference laboratories [2,81]; this can translate into significant delays in obtaining results, explaining why therapy in the form of TPE is initiated empirically to minimize possible morbidity and associated mortality [82]. This represents a major limitation, indicating that, even when available in-house, and despite its cost effectiveness [83], if not routinely performed to retain staff competency in running the test, it can still result in delayed disease recognition or the unnecessary transferring of patients to alternative facilities [84]. Once test results are positive, an inhibitory (Bethesda) assay is performed to establish the nature of the antibody present [85]. This consists of testing for the presence of neutralizing autoantibodies using mixing studies of heat-inactivated patient and normal plasma, usually in a 1:1 concentration or through pre-established serial dilutions [86]. In this way, a percentage inhibition can be obtained and resulted as antibody inhibitory units [87]. However, non-inhibitory antibodies will be missed in this manner.

More recently, an enzyme-linked immunosorbent assays (ELISA) antibody assay has been developed and is available at certain reference laboratories. This assay, which captures IgG antibodies on an ADAMTS13-coated plate, detects all antibodies against ADAMTS13 regardless of whether they are inhibitory or non-inhibitory [79]. This makes it more sensitive for iTTP but less specific in cases where patients have non-neutralizing antibodies but no disease. When combined with a low ADAMTS13 activity level, and in the right clinical context, the combination of a negative inhibitory (Bethesda) assay and a positive antibody (ELISA) assay may indicate iTTP caused by non-neutralizing antibodies. Thankfully, commercially available ELISA kits have become available which quantify the ADAMTS13 antigen [85,88]. These can possibly shorten the duration of obtaining test results, especially when non-neutralizing antibodies are present [89]; these results would not be, however, indicative of the functional state of a given ADAMTS13 protein concentration [90]. Nevertheless, the accessibility of these ELISAs would allow for stronger laboratory evidence that there is an enzyme deficiency, thus triggering therapy initiation [85].

However, despite these options for ADAMTS13 testing being currently available, it still remains to be determined how frequently ADAMTS13 activity tests should be performed once patients enter remission, especially since there is still a lack of consensus on which activity level should trigger the reinitiation of interventions/therapies [82,91]. As mentioned earlier, ELISA availability for diagnosis is a major step forward; however, in order to generate a more expeditious turnaround time, testing platforms should be automated so that testing variations and/or human error can be minimized [92]. Furthermore, ADAMTS13 activity can be a short-lived state in the setting of high or rapidly increasing antibody titers; thus, likely testing results would need to be considered in the context of a down-trending platelet count and, as a result, autoantibody levels and/or their specificity may be a better gauge of therapy initiation [75]. More importantly, ADAMTS13 activity testing is uninformative in regard to the conformational status of the enzyme at the time of antigen measurement which would be fundamental in differentiating cTTP from iTTP, and when the recovery phase from the disease begins [93,94]. Along these lines, a newly developed test that utilizes fiber optic surface plasmon resonance (FO-SPR) has been reported to specifically detect the closed or open conformations of ADAMTS13 in healthy and acute-onset iTTP patients, respectively, not only with a short turnaround time of 3 h but, above all, with high correlation with the FRETS-VWF73 assay results [93]. Similarly, development of a detection test for autoantibodies to ADAMTS13 also taking advantage of the FO-SPR methodology has yielded a highly sensitive and specific platform that gives results in as little as 2.5 h, which is half the time of a regular ELISA assay [95]. The major advantage of this assay is that it can be adapted to changing antibody specificities and possibly modified so that it can be used for a number of autoimmune diseases. These new approaches hold promise that testing in the near future can be more specific, providing timely information in relation to molecular ADAMTS13 conformation and autoantibody specificity.

## 7. Potential Role of Antibodies in Bone Marrow Suppression

An interesting possibility has emerged from data reported by our group over the last decade that is suggestive of additional disease mediators. Even though ADAMTS13 deficiency occurs uniformly across patients, we have also observed with a high degree of specificity that all patients presenting with new-onset iTTP have a suppressed immature platelet response below the reference range for this variable in both adults and children [91,96]. This is paradoxical, since the removal of platelets from circulation to form microthrombi should elicit a marked increase in the immature platelet output at the level of the bone marrow [97]; however, instead there is an almost absolute suppression in this production. This suppressive phenomenon is promptly reversed by the initiation of TPE, and these increases in immature platelet counts translate into corresponding increases in the mature platelet count compartment on average 1–2 days later [98]. This would not be surprising, since other diseases have been shown to require two hits or more in order to manifest themselves. Potentially, this suppression may be in response to a series of inhibitory molecules, but one of these could represent a different autoantibody with a specificity yet to be defined. This should be of no surprise, since the development of an autoantibody does not preclude the organism from making additional autoantibodies to other antigens. This is currently a major focus of investigation.

## 8. Working Antibody-Mediated Disease Model and Therapeutic Options

Based on what has been presented, it cannot be disputed that iTTP is a rare and severe clinical presentation; however, a better understanding of disease pathology, including ADAMTS13 deficiency, has led to a more timely recognition and initiation of targeted therapy. Decreased ADAMTS13 activity leads to a buildup of the ultra large vWF multimers along endothelial walls, resulting in platelet-rich microthrombi and the characteristic triad of thrombocytopenia, hemolytic anemia, and end-organ ischemia characterizing the disease. Like other autoimmune diseases, the formation of autoantibodies in iTTP is likely caused by immune system dysregulation and dysfunction, being mediated by a combination of genetic predisposition and environmental factors. Thus, post-infectious molecular mimicry in genetically susceptible individuals is potentially the most likely etiology of disease. IgG antibodies are the dominant antibody class in the plasma of patients with acute iTTP, and the levels of autoantibodies have generally been found to positively correlate with iTTP disease severity, mortality, and relapse risk.

Anti-ADAMTS13 autoantibodies are polyclonal in nature, and they have been found targeting all ADAMTS13 domains at one point or another; nevertheless, the central cysteine-rich spacer region appears to be the set of epitopes that is highly significant in the development of iTTP. For this reason, most patients with iTTP will produce anti-cysteine-spacer antibodies—even if other antibodies may be present as well—and this domain is known to possess enzymatic activity over ultra large vWF multimers. This argues that therapeutic agents that target or block these specific domains can prove beneficial to patients. This is similar to the mechanism by which caplacizumab, the only TTP-specific therapeutic agent, binds to the A1 domain of vWF, thus stopping the chain of events that leads to platelet binding and subsequent thrombocytopenia [99,100]. Nevertheless, despite the usefulness of this new therapeutic agent, this does not prevent the enzyme from assuming the immunogenically active open conformation that remains as the main driver in autoantibody formation, nor does it prevent autoantibody formation.

Anti-ADAMTS13 antibodies exert their effects either via inhibitory or non-inhibitory mechanisms. As discussed, neutralizing antibodies disable the enzyme’s proteolytic activity, while non-neutralizing antibodies result in depleted enzyme levels, specifically, by the formation of antigen–antibody immune complexes that are cleared from circulation. Since the binding of antibodies is highly conformation-dependent and increases when ADAMTS13 is in the “open” conformation, development of agents that block this open conformation or reduce its presence will be therapeutic. Moreover, since autoantibodies can also be responsible for inappropriate conversion of the enzyme from the “closed” to “open” conformation in the absence of endothelial injury, those above-mentioned therapeutic agents can prove beneficial in this subgroup of patients.

As alluded to previously, control of autoantibody concentration and production is essential to mediate recovery. For this purpose, TPE has been used effectively for decades to remove the offending antibodies while supplementing the enzyme; however, it does this in a non-specific way. Furthermore, the use of steroids and the anti-CD20 antibody rituximab have become mainstay in the therapy repertoire to treat iTTP patients not only in the acute phase but also in patients prone to relapse to depress antibody production [101]. Even in cases when there is lack of response to rituximab, other currently available anti-CD20 monoclonal antibodies such as obinutuzumab and ofatumumab have proven effective in eliciting responses in patients shown to be refractory or allergic to rituximab [102,103]. Similarly, despite these advances, there needs to be better restoration of enzyme activity to physiologic levels, so the development of recombinant ADAMTS13 represents a new age in the treatment of patients [104]. One version of this reagent, termed Adzynma, received recent approval by the United States’ Food and Drug Administration for the treatment of patients with cTTP [105]. This medication can be given intravenously even daily as needed, and, in instances of high autoantibody titers, it can be given in such doses that it can fully restore ADAMTS13 in iTTP patients [106]. Other groups are currently working on developing genetically modified ADAMTS13 with protein residue alterations that resist the binding of autoantibodies while retaining overall enzymatic function [107]. Finally, a clear understanding of the kinetics driving vWF conformational changes from “closed” to “open” in iTTP will be necessary to develop pharmacological reagents that either prevent it or minimize it during an acute attack. Such reagents will likely be of major utility in patients on remission to stop vWF from opening and re-stimulating the immune system with the epitopes that led to the initial autoantibody production.

## 9. Conclusions

In attempting to summarize what is known up to now about the pathophysiology of iTTP antibodies, one overarching explanation proposes a kind of positive cooperativity among polyclonal antibodies [108]. According to this model, a “synergistic effect” exists by which non-inhibitory antibodies, such as those targeting the distal C-terminal domain, induce an allosteric conformational change in ADAMTS13 from the “closed” to the less stable but enzymatically active “open” state. Adoption of this “open” conformation “modulates its inhibition”, possibly by revealing the existence of cryptic epitopes in the cysteine-rich spacer domain. Inhibitory antibodies targeting the spacer domain are then free to bind to the cryptic epitopes, which decreases levels of functionally active ADAMTS13. While promising, and adding to the current knowledge on the disease, this model requires further refinement in order to guide research and the discovery of effective therapies, diagnostics, and guidelines for iTTP management. In the coming years, strengthening our understanding of the structure, function, and effects of anti-ADAMTS13 antibodies will be needed to address and nullify the high morbidity and mortality associated with this disease.

## Data Availability

All relevant data are included in the manuscript.
